# Supplementary material for: Bimodal Peptide Collision Cross Section Distribution Reflects Two Stable Conformations in the Gas Phase
Source: J Proteome Res. 2026 Apr 29;25(6):2813–25. doi: 10.1021/acs.jproteome.5c01159 (PMC13247972; doi:10.1021/acs.jproteome.5c01159)
Supplement: Supplementary file 1 [file pr5c01159_si_001.pdf]

Supplementary Information for:

Bimodal peptide collision cross section distribution reflects two stable conformations in the gas phase

Juan Restrepo[1,3,#], Daniel Szoelloesi[2,#], Tobias Kiermeyer[1], Christoph Wichmann[1,3,*], Helmut Grubmüller[2,*] and Jürgen Cox[1,*]

[1]Computational Systems Biochemistry Research Group, Max-Planck Institute of Biochemistry, Am Klopferspitz 18, 82152 Martinsried, Germany.

[2]Department of Theoretical and Computational Biophysics, Max Planck Institute for Multidisciplinary Science, Am Fassberg 11, 37077 Göttingen, Germany

[3]Current address: Aplusia GmbH, Türkenstr. 5, 80333 Munich

[#]These authors contributed equally to the publication.

[*]Correspondence: cox@biochem.mpg.de, hgrubmu@gwdg.de, christoph@aplusia.com

Table of Contents:

Figure S1: Fit to bivariate Gaussian per protease and charge S2

Figure S2: Mobility-m/z distribution per organism and charge S3

Figure S3: Mobility-m/z distribution per protease and charge S4

Figure S4: Reduced mobility versus m/z for two enzymatic groups S5

Figure S5: Intensity profile on the mobility dimension for a precursor S6

Figure S6: Potential energy histogram of globular and helical conformations S7

Figure S7: Geometric fit and error distribution for each charge state S8

Figure S8: Selected transversal slices with constant CCS S9

Figure S9: Distribution of energies of peptide001in different configurations S10

Figure S1: Fit to bivariate Gaussian per protease and charge

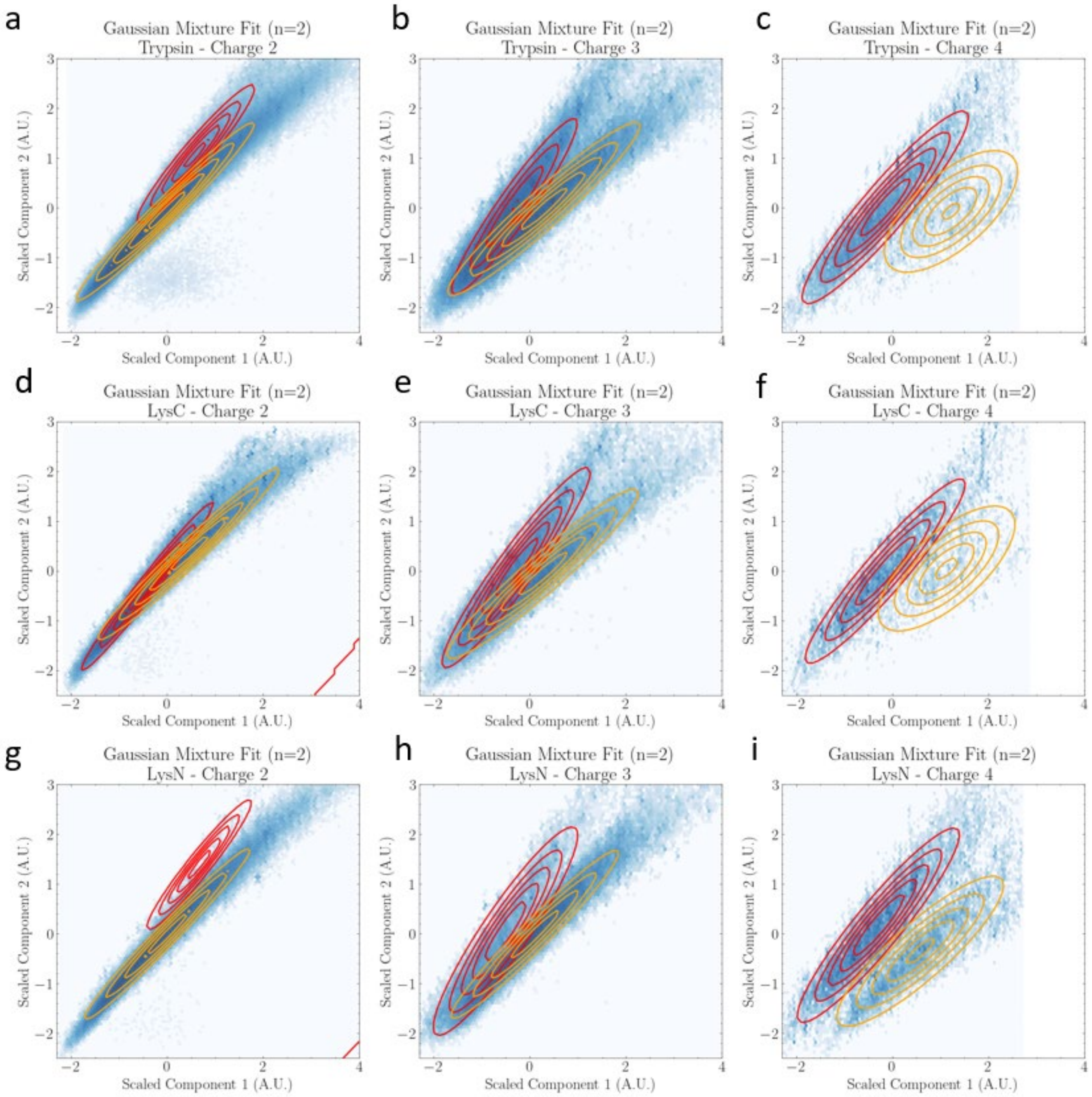

Fit to the sum of two bivariate normal distributions per protease and charge state overlaid on the corresponding distribution. The distributions are normalized to have zero mean and unit variance.

Figure S2: Mobility-m/z distribution per organism and charge

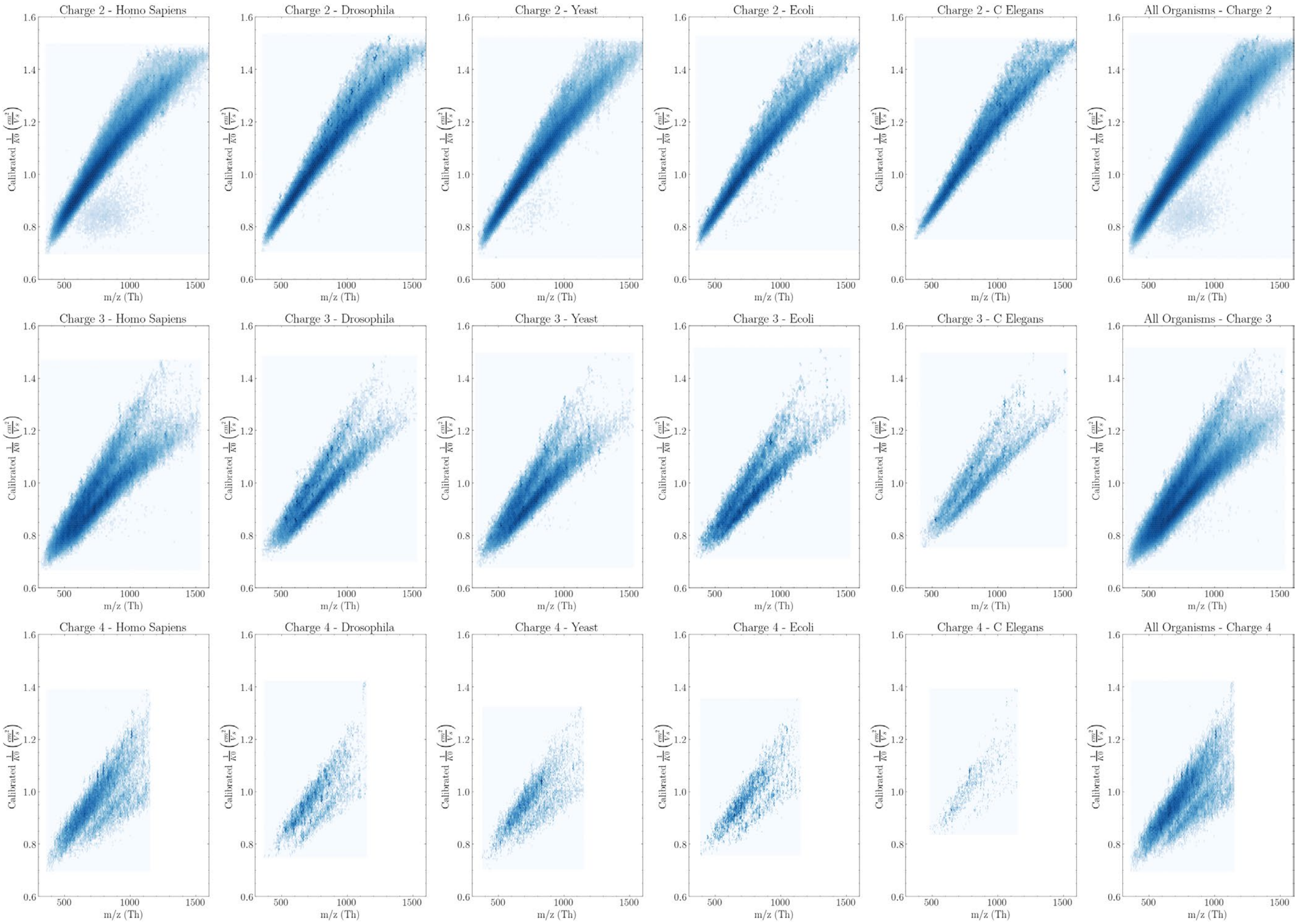

Distribution of peptides in the space of reduced mobility calibrated across all runs versus mass-to-charge ratio for all charges and organisms.

Figure S3: Mobility-m/z distribution per protease and charge

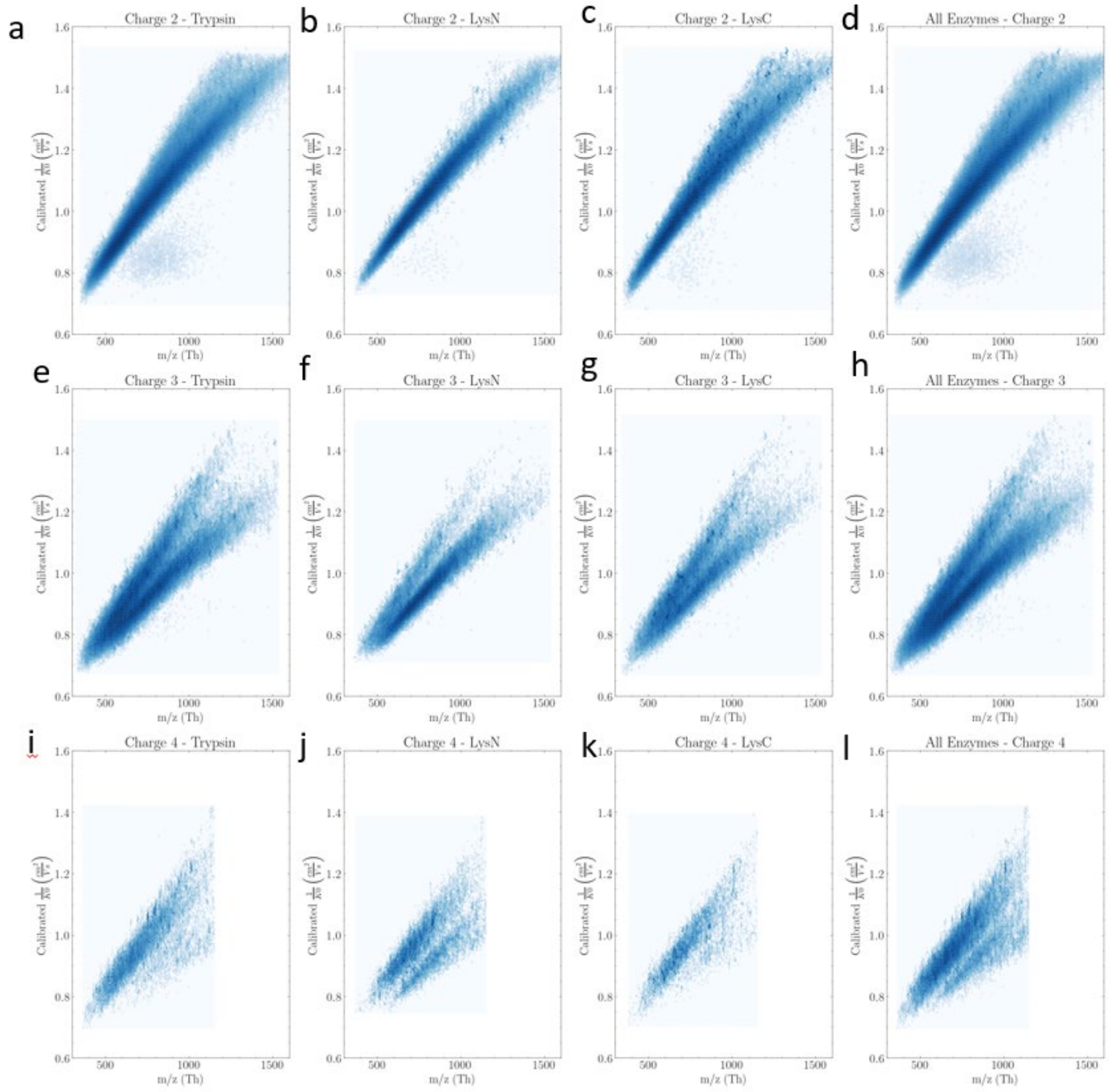

Distribution of peptides in the space of reduced mobility calibrated across all runs versus mass-to-charge ratio for all charges and proteases.

Figure S4: Reduced mobility versus m/z for two enzymatic groups

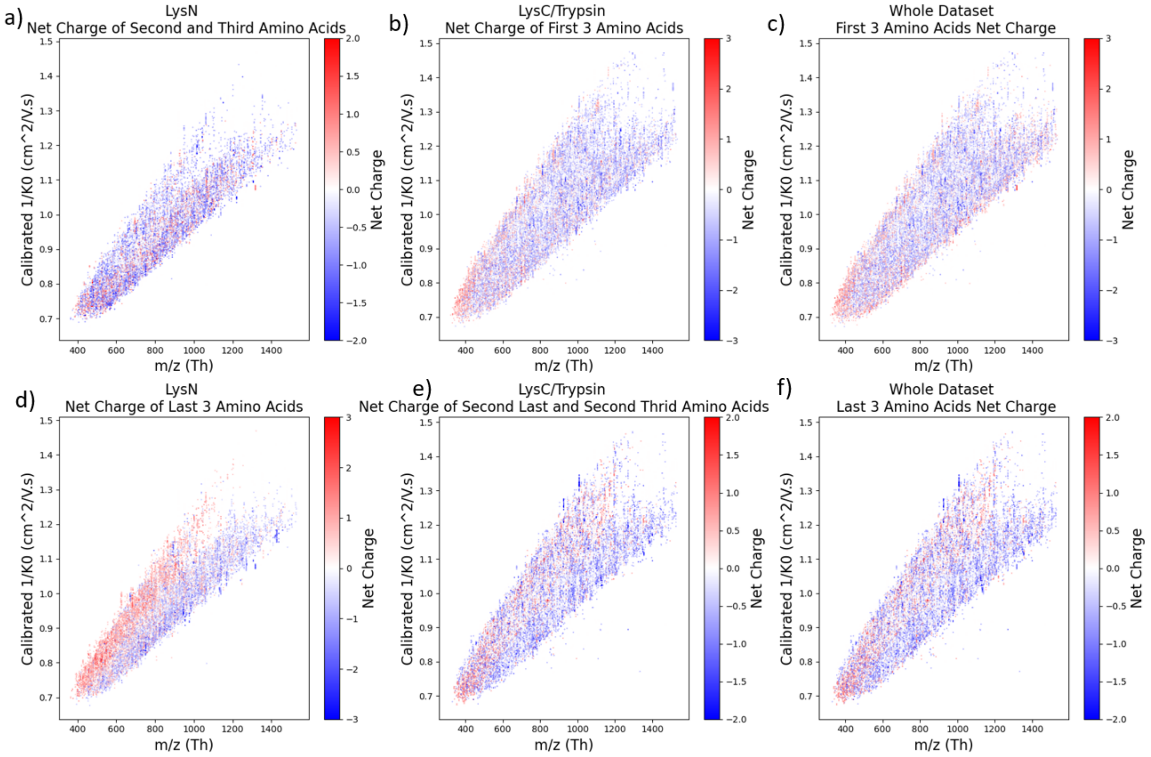

Distribution of peptides in the space of reduced mobility calibrated across all runs versus mass-to-charge ratio for two enzymatic groups: LysC/Trypsin and LysN. Each distribution is colored by net charge in either the first three or the last three amino acids ignoring the charge associated to the protease. The distribution of the total dataset without enzymatic division is also shown on the right-most column.

Figure S5: Intensity profile on the mobility dimension for a precursor

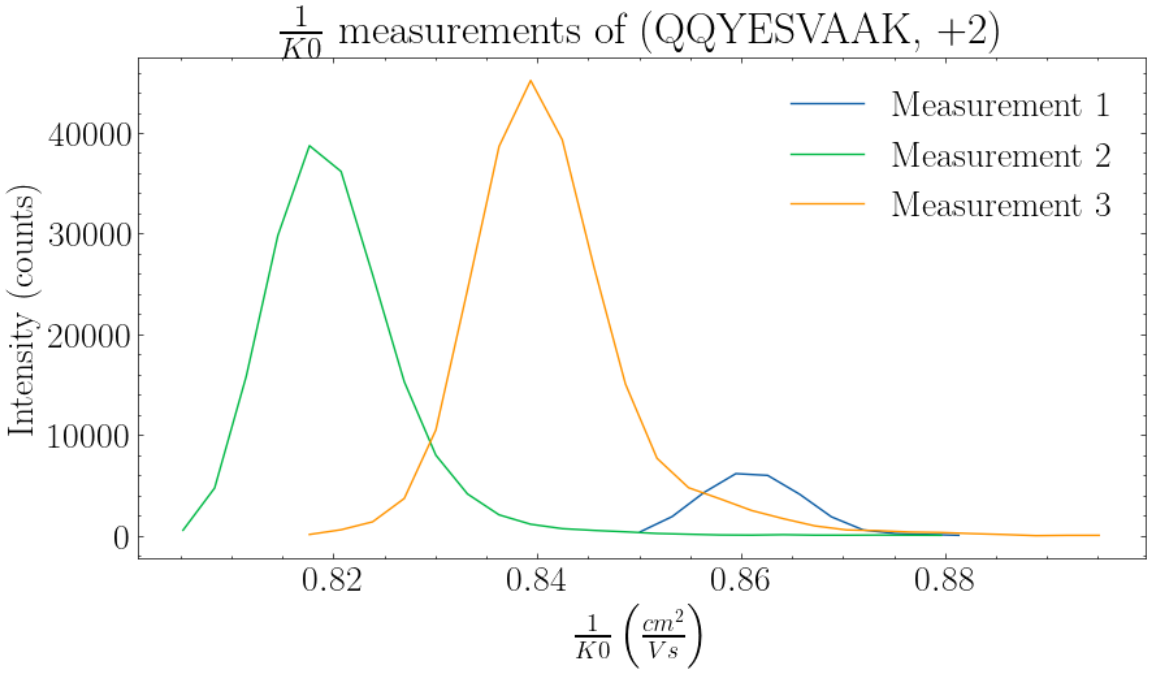

Intensity profile on the reduced mobility dimension for a particular precursor (see title) that exhibits multiple peaks.

Figure S6: Potential energy histogram of globular and helical conformations

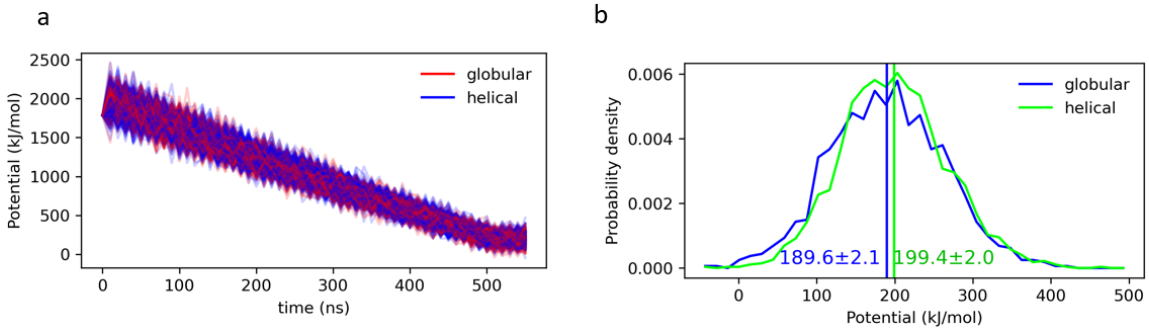

Potential energy histogram of globular and helical conformations of the P1 peptide. Vertical lines and label indicate the mean potential energy and its standard error. The last 40 ns of the quenching simulations at constant temperature 305 K were used for this analysis. Grouping into globular (blue) and helical (green) was based on predicted CCS for the final conformation, structures above 850 Å2 were considered helical, otherwise globular.

Figure S7: Geometric fit and error distribution for each charge state

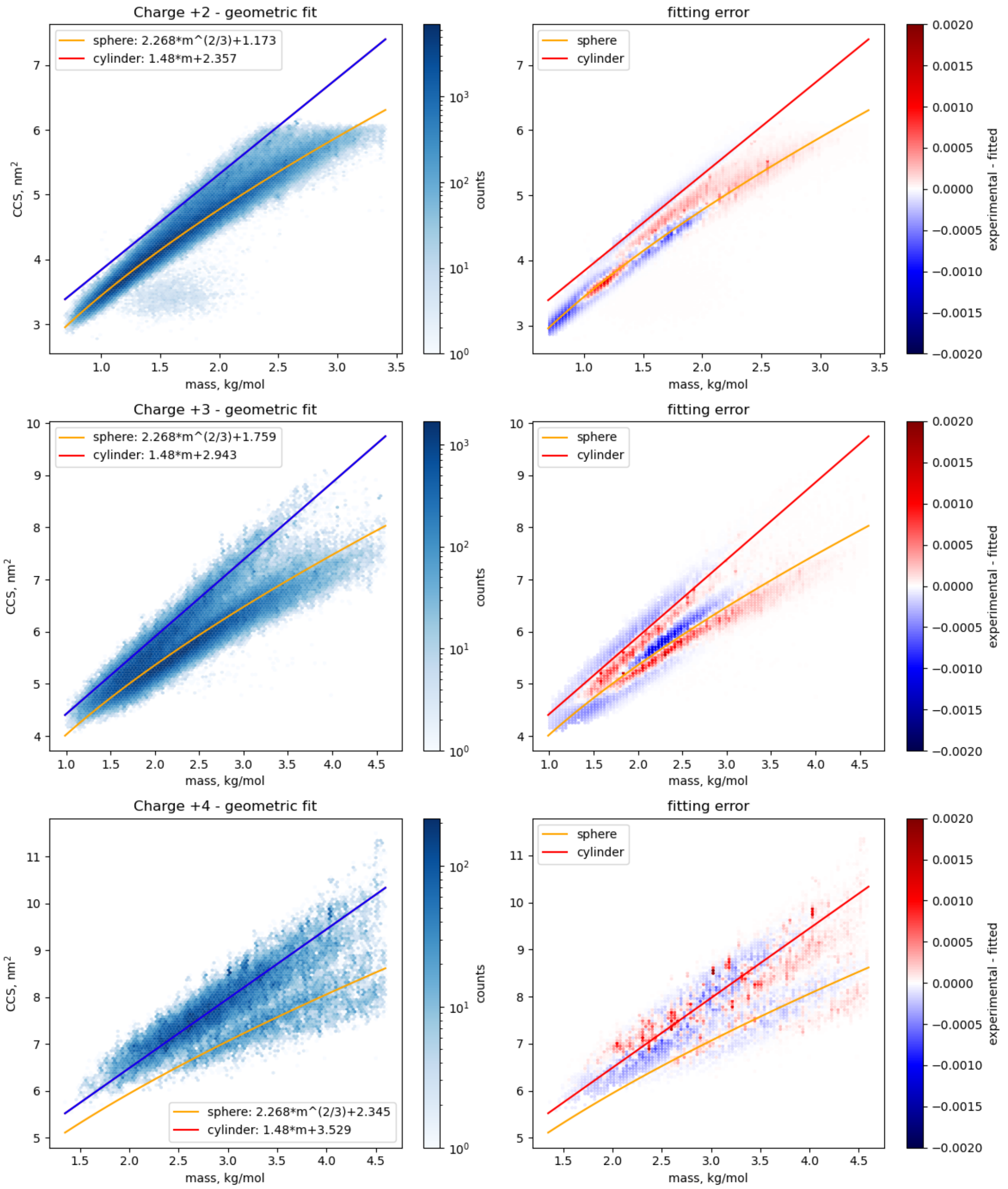

Geometric fit (left column) and fitting error distribution (right column) for each charge state.

Figure S8: Selected transversal slices with constant CCS

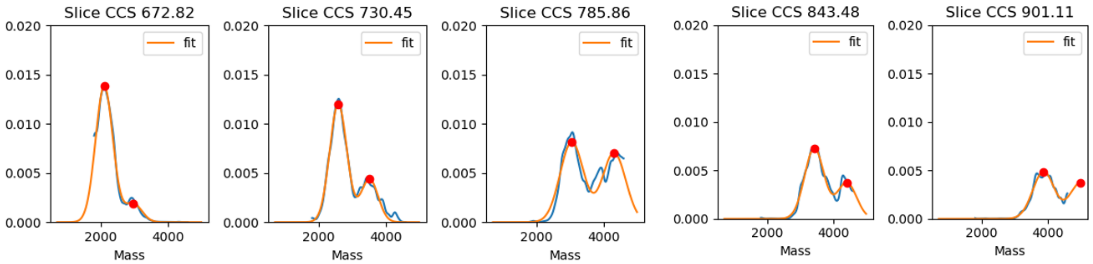

Selected transversal slices with constant CCS over the mass dimension(blue line) for charge state four together with the fit to the sum of two one-dimensional gaussian distributions (orange line).

Figure S9: Distribution of energies of peptide001in different configurations

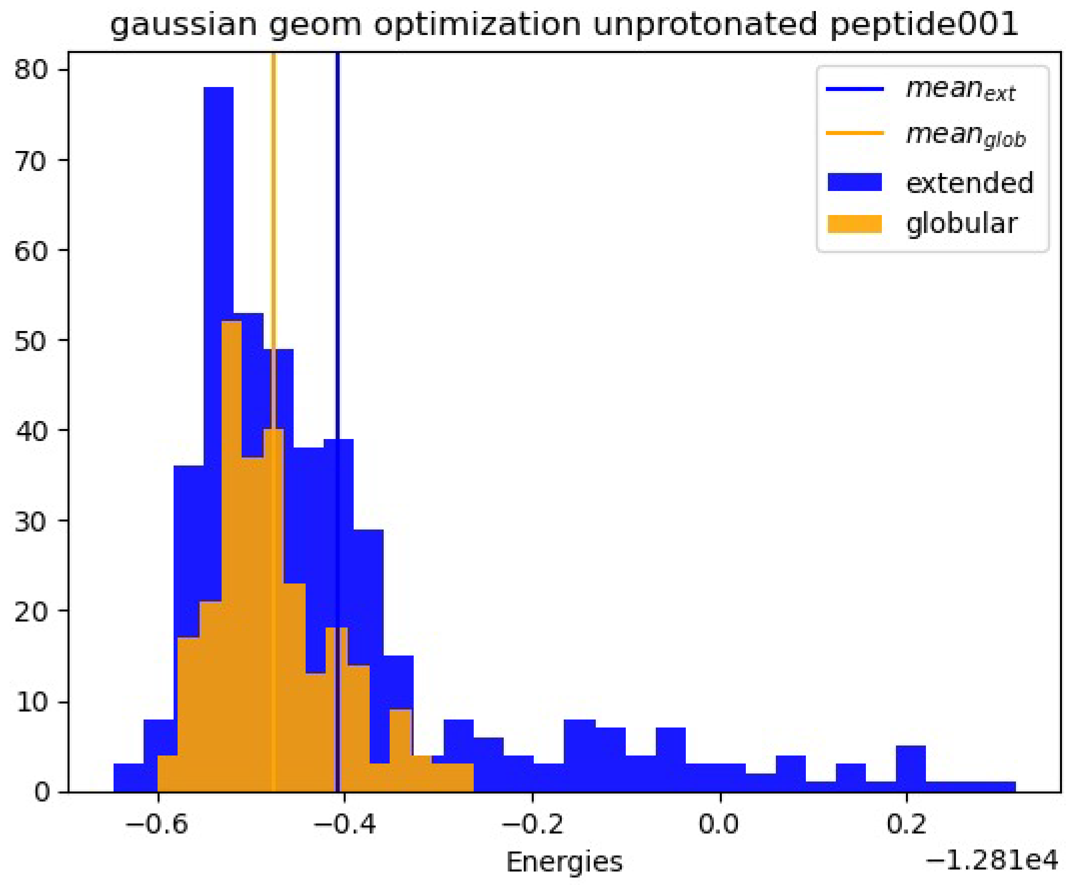

Distribution of energies of peptide001(see table 1) in different configurations obtained by simulations. The extended or globular label was set by observation of the CCS distribution of the simulated peptides. The energies were calculated using the Gaussian software.
